# Role of the AP2 β-Appendage Hub in Recruiting Partners for Clathrin-Coated Vesicle Assembly

**DOI:** 10.1371/journal.pbio.0040262

**Published:** 2006-08-15

**Authors:** Eva M Schmid, Marijn G. J Ford, Anne Burtey, Gerrit J. K Praefcke, Sew-Yeu Peak-Chew, Ian G Mills, Alexandre Benmerah, Harvey T McMahon

**Affiliations:** 1 Medical Research Council Laboratory of Molecular Biology, Cambridge, United Kingdom; 2 Department of Infectious Diseases, Institut Cochin, Paris, France; 3 Center for Molecular Medicine Cologne, Institute for Genetics, University of Cologne, Köln, Germany; 4 The Oncology Department, University of Cambridge Hutchison/MRC Cancer Research Centre, Cambridge, United Kingdom; Princeton University, United States of America

## Abstract

Adaptor protein complex 2 α and β-appendage domains act as hubs for the assembly of accessory protein networks involved in clathrin-coated vesicle formation. We identify a large repertoire of β-appendage interactors by mass spectrometry. These interact with two distinct ligand interaction sites on the β-appendage (the “top” and “side” sites) that bind motifs distinct from those previously identified on the α-appendage. We solved the structure of the β-appendage with a peptide from the accessory protein Eps15 bound to the side site and with a peptide from the accessory cargo adaptor β-arrestin bound to the top site. We show that accessory proteins can bind simultaneously to multiple appendages, allowing these to cooperate in enhancing ligand avidities that appear to be irreversible in vitro. We now propose that clathrin, which interacts with the β-appendage, achieves ligand displacement in vivo by self-polymerisation as the coated pit matures. This changes the interaction environment from liquid-phase, affinity-driven interactions, to interactions driven by solid-phase stability (“matricity”). Accessory proteins that interact solely with the appendages are thereby displaced to areas of the coated pit where clathrin has not yet polymerised. However, proteins such as β-arrestin (non-visual arrestin) and autosomal recessive hypercholesterolemia protein, which have direct clathrin interactions, will remain in the coated pits with their interacting receptors.

## Abstract 2

Formation of clathrin-coated vesicles, important in endocytosis, relies on accessory proteins assembled by adaptor protein complex 2 (AP2). Here, mass spectrometry and crystallization identifies proteins recruited by AP2's β-appendage for this purpose.

## Introduction

Clathrin-mediated endocytosis is the process whereby cargo molecules are selected for incorporation into vesicles surrounded by a coat protein, clathrin. “Accessory proteins” organise the process of coat assembly and disassembly as well as the process of membrane budding and fission [1–5]. Some of these accessory proteins act as “cargo adaptors” and help select the cargo content of the nascent vesicle. In synaptic vesicles there are over ten different transmembrane cargo components, each in their constant stoichiometries and packaged in vesicles of uniform size to ensure reliable synaptic transmission [6,7]. This is a remarkable achievement of the clathrin-adaptor machinery. Thus we are seeking an understanding of the biogenesis of the synaptic vesicle organelle (and indeed clathrin-coated vesicles in general) and the principles of how accessory cargo adaptors and other accessory proteins are organised to achieve vesicle formation.

**Figure 1:**
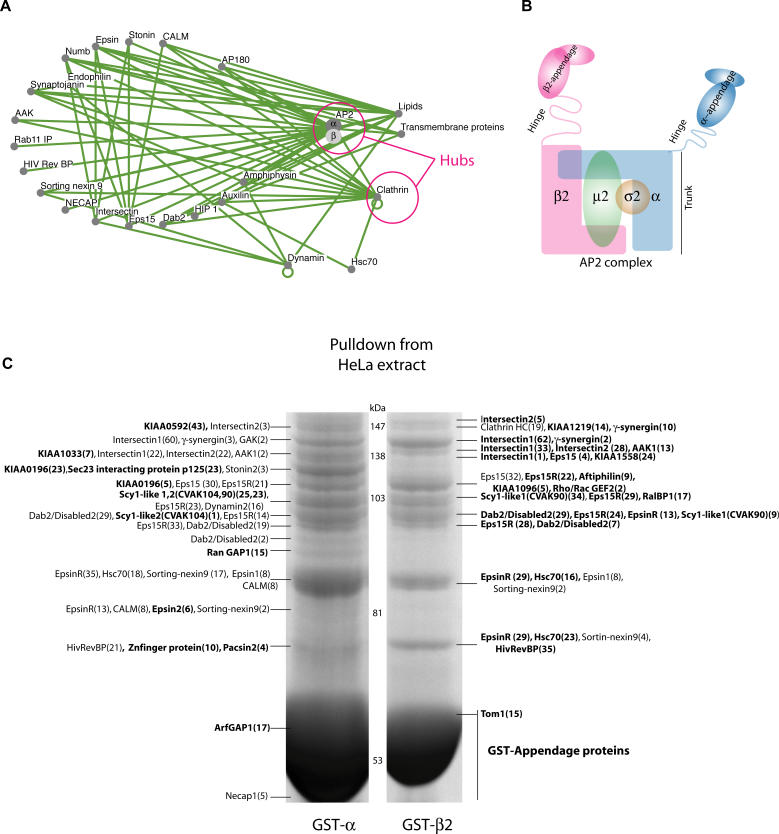
New Protein Interaction Partners for the α- and β-Appendage Domains of AP2 Adaptors (A) Plot of the network of protein interactions in clathrin-mediated endocytosis. AP2 adaptors and clathrin have disproportionately large numbers of interactors and so are the hubs of this network. Dynamin is a “party” hub as it is shared between different networks but we have not included all its interactors. (B) Scheme of AP2 showing the overall domain architecture and the appendages where most of the protein interactors bind are located on flexible linkers called “hinges.” (C) Protein interactors of α- and β2 appendages from HeLa cells as determined by LC-MS/MS of Coomassie stained bands. Bolded proteins were not detected previously. The interaction of CVAK104 and CVAK90 were tested and confirmed by yeast-2-hybrid analysis. The numbers of peptides sequenced from each protein are given in brackets. Further mass spectrometry data from brain and liver samples, accession numbers, domain structures and details are given in Figures S1 and S2.

Clathrin-coated vesicle (CCV) formation is frequently viewed as a linear series of steps culminating in the detachment of a vesicle from the parent membrane. However the initial steps of cargo concentration, membrane invagination and coat assembly likely occur in parallel and, to achieve this, many proteins must work together [8]. For proteins involved in the various aspects of CCV formation this cooperation necessitates many interconnections and the process is best described as a dynamic network. When we plot the characterised possible protein interactions in endocytosis, ignoring the competition and spatial constraints, we obtain an “endocytic interactome” (see scheme, Figure 1). In the endocytic interactome some proteins have disproportionately large numbers of interaction partners and these are classified as “hubs” [9]. The tetrameric adaptor protein complex 2 (AP2) and clathrin are examples of such hubs in this network.

There are four AP complexes used in different trafficking pathways (AP1–AP4) each having two large subunits (adaptins: γ, α, δ, ɛ, and β1–4) and two smaller subunits (μ1–4 and σ1–4; see reviews above and schematic of AP2 in Figure 1B). The core domain (trunk) of each complex interacts with both PtdInsPs and cargo (predominantly via the μ and α-subunits) thereby anchoring it to membranes [10]. The C-terminal “appendage domains” of the large subunits recruit interaction partners from the cytosol facilitating their concentration at sites of coated pit formation. Appendage domains are found at the ends of flexible linkers (hinge domains) that extend from the core of the adaptor complex. This provides the opportunity to search a wide area of surrounding cytoplasm for accessory proteins including other cargo adaptors (see http://www.endocytosis.org/Adaptors/Appendages.html for structural aspects).

In our previous work we investigated how the α-appendage interacts with its many binding partners [8] and found that some proteins interact tightly with isolated α-appendage domains while others interact more effectively when appendages are concentrated, as occurs during clathrin-coated pit (CCP) formation. Each α-appendage has at least two distinct interaction sites for short linear peptide motifs (see also [11]). If a single interaction partner occupies both sites then that allows interactions with a higher avidity (a value that is between the sum and the product of the affinities for each site). Given that the multiple peptide motifs are generally found in unstructured domains, this provides a mechanism for generating high avidity and readily reversible interactions. We proposed that during coated pit assembly many different accessory proteins will interact simultaneously with the AP2 hub (made up of concentrated AP2s) but in stoichiometries related to their affinities and concentrations. In this view the AP2 α-appendage is not initially a hub in solution but has the properties of a hub only during CCP formation, and again rapidly loses these properties when clathrin is recruited and polymerised. Thus AP2 is a hub only at initial stages of network assembly and these AP2 hub assembly zones are found at the leading edges of CCP formation [8].

In this paper we are interested in the organisation of the endocytic interactome and therefore in the repertoire of AP2 interactors in brain and non-brain tissues and by extension in synaptic vesicle endocytosis and receptor-mediated endocytosis. We find that in an AP2 complex the β2-appendage works in partnership with the α-appendage to bind a similar but not identical subset of appendage ligands, thereby extending the interaction repertoire. The β-appendage collaborates with the α-appendage in pulling partners to sites of pit assembly. The β-appendage has an additional function as a unique clathrin assembly platform [12,13] that results in the displacement of β-ligands despite the low affinity of the appendage for clathrin. This ensures the coated pit matures from an initial assembly zone to a clathrin-coated pit. By structural analysis we elucidate the modes of ligand interactions with the β-appendage. We speculate on how the relative affinities and avidities of accessory proteins are superseded by the matrix interaction of polymerised clathrin. We see the process gradually moving from liquid-phase dynamics in the cytoplasm, through membrane-anchored dynamics to more solid-phase dynamics as clathrin polymerises around the nascent vesicle. Here we refer to the protein interactions in this maturation as moving from affinity (as measured by the simple interactions of one protein with another), through avidity (the interaction of clustered components with multivalent ligands) to “matricity” (the interaction of a matrix with its environment).

## Results

### Ligands of the β-Appendage

The AP2 adaptor complex with its two appendages acts as an interaction hub for numerous accessory proteins, whether these be accessory cargo adaptors or accessory proteins involved in the mechanics of CCV formation. We have previously carried out chromatography tandem mass spectrometry (LC-MS/MS) analysis for α-appendage ligands in brain extract [8] and γ-appendage ligands in brain extracts have been analysed by the group of Robinson [14]. We have extended this using glutathione S-transferase (GST) α and β2 appendages coupled to agarose beads in HeLa, brain and liver extracts (Figure 1C and Figure S1), and analyse the protein interactors with LC-MS/MS. Over 20 different proteins are found in the β2-appendage pull-down from HeLa cell extracts (Figure 1C). These include clathrin, intersectin1 and 2, γ-synergin, AAK, Eps15, Eps15R, aftiphilin, Dab2/Disabled2, epsin and epsinR, RalBP1, Hsc70, sorting-nexin9 (snx9), HivRevBP, and Tom1, along with some proteins not yet named (KIAA1219, KIAA1558, KIAA1096), and a kinase like protein called Scy1-like1. Many of these have not been observed before (highlighted in bold in Figure 1C). There is a surprising overlap of binding partners with the α-appendage, but there are also unique protein interactors for each appendage. In the HeLa cell pull-down 25% of the β2 interactors are not found on α. We have also found some new interaction partners for the α-appendage not detected in our previous analysis of α-ligands in brain extract (including epsin2, pacsin/syndapin2, Scy1-like1 and Scy1-like2, Sec23 interacting protein p125, RanGAP1, Znfinger protein, and ArfGAP1), along with some proteins not yet named (KIAA0592, KIAA1033, KIAA0196). This might reflect the greater abundance of some of these proteins in HeLa cells. KIAA1414 was found in the rat brain β2-appendage pull-down (Figures S1 and 2) and this has previously been noted as a component of CCVs [15]. KIAA0685, a SAPS domain containing protein, is also found in rat brain pull-downs and is related to KIAA1558 found in the HeLa cell pull-downs.

The pull-down experiments do not prove that all of these newly identified interactors are direct. However the majority of interactors contain domains that are predicted to be largely unstructured and by sequence analysis contain multiple repeats of the type that we already know interact with α-appendages (we call these “motif domains”, see also Figure S2). One protein that we find in α- and β-appendage pull-downs from HeLa, liver and brain extracts is Scy1-like1. This is a distant homologue of Scy1-like2 which is found only in α-appendage pull-downs (Figure 1C). There is 16% amino acid identity between Scy1-like1 and 2. Scy1-like2 was previously found in CCVs and was thus renamed as clathrin-coated vesicle associated kinase of 104kDa (CVAK104)[16]. Scy1-like1 contains multiple DxF motifs of the type that bind to the top site of the α-appendage and thus is likely to function in CCV formation. To retain nomenclature we call it CVAK90 (as the longest human splice form is predicted to be 90kDa). We verified a direct interaction of CVAK104 and CVAK90 with both the α- and β-appendages using yeast-2-hybrid (unpublished data).

Our pull-down experiments so far have examined the interaction partners of appendages from the AP2 complex. The AP1 complex has γ- and β1-appendage domains. β1- and β2-appendages share over 70% amino acid identity and in our experiments they interact with a similar range of proteins (Figure 1C and Figure S1B). Previously reported γ-appendage interactors like γ-synergin and aftiphilin [17–21] also bind to β-appendages but not strongly to the α-appendage in our pull-downs. Thus they will form stronger networks with γ and β1 and therefore get selected for AP1-CCV formation.

We see from this data that the accessory cargo adaptor Dab2/Disabled2 (a cargo adaptor for proteins like megalin) binds to both α and β appendages, and it is likely that some of the other accessory proteins that bind to both α and β appendages will also have cargo adaptor status. Thus a view that accessory cargo adaptors interact exclusively with the β-appendage cannot be generalised. Nevertheless some accessory adaptors like β-arrestin (for internalisation of G-protein coupled receptors [GPCRs]) and autosomal recessive hypercholesterolemia protein (ARH; for internalization of LDL receptors) bind to the β-appendage alone [22–24]. We do not detect them here, presumably because of the low abundance of these proteins in HeLa cells.

The few differences in the major ligands found in brain extracts compared with HeLa cell extracts (Figure 1C and Figure S1) show that CCV formation in the brain is not so extremely specialised. Nevertheless we do find differences, like the abundance of amphiphysin and AP180 in α-pull-downs from brain, compared with sorting-nexin9 and CALM in non-neuronal tissues.

Many of the α-appendage binding partners were also β-partners and thus there is not the degree of protein selectivity between the appendages we had expected. Since two different appendages appear to be necessary we need to understand why, and thus we investigate the modes of ligand interactions.

### Two Interaction Sites for New Motifs on the β-Appendage

**Figure 2:**
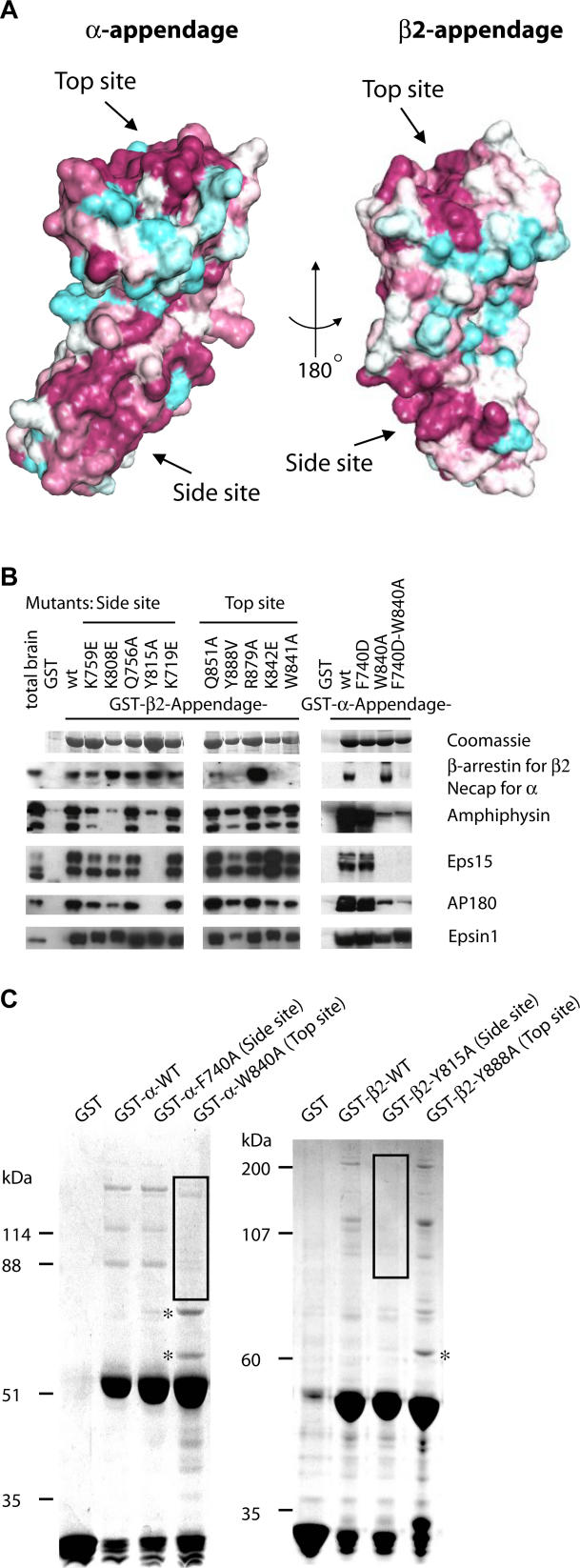
The β2-Appendage Has Top and Side Sites for Protein Interactions (A) Surface residues that are conserved from yeast to man are coloured from maroon (highly conserved) through white to sky-blue (least conserved). The α-appendage is shown for comparison. Both have two conserved patches, a top site and a side site. (B) Mutants of top and side sites in β compared with mutants of α. GST wild-type and mutant appendages were used in pull-downs from brain extract. β-arrestin binds specifically to the top site of β2, while most other ligands appear to bind more tightly to the side site. (C) Coomassie gel analysis of protein interactors with α- and β2-appendages from brain extracts. The boxed areas show the most effective mutants at displacing ligands. Asterisks mark positions of co-purified chaperones.

Protein-protein interaction surfaces are more highly conserved than surrounding areas. We have used this conservation of surface residues in our previous analysis of the α-appendage to predict binding sites and now extend the same analysis to the β-appendage using sequences extending from yeast to man. There is a conserved site on the platform sub-domain of the appendage (top site) and on the side of the β-sandwich sub-domain (side site, Figure 2A). The top site is in a comparable position in α and β2 (for mutagenesis see [13]) but the side site is almost on the opposite side of the β-sandwich compared to the corresponding side site of α.

We made critical point mutations of the interaction sites and looked at a variety of ligands using pull-downs and Western blotting. Mutation of a tyrosine on the side site (Y815A) of the β2-appendage was highly effective at displacing amphiphysin, Eps15 and AP180 (Figure 2B). Mutation of surrounding lysines to glutamates also weakened these protein interactions. β-Arrestins have previously been proposed to bind specifically to the β-appendage and are not displaced by these side site mutants. Whereas β-arrestin is completely displaced by the top site mutant Y888V, amphiphysin, Eps15, AP180, and epsin1 are only weakened by this same mutation. Thus these ligands must have interaction motifs for both the top site and the side site. Two interaction sites were also found in a recent publication [25] but the authors believe these sites are independent. Several top site mutants showed a stronger interaction with ligands. While R879A binds with a higher affinity to β-arrestin, K842E binds with a higher affinity to Eps15 and a lower affinity to AP180 and amphiphysin. From these mutants we conclude that this top site has several different modes of interaction and this fits with the extensive nature of the conserved interaction surface.

A comparison of Coomassie-stained interactors of the main mutants of top and side sites in both α and β2 appendages (Figure 2C) shows that the main ligand interaction site on α is the top while the main interaction site on β is the side.

### Multiple Interaction Sites Lead to Increased Avidity

**Figure 3:**
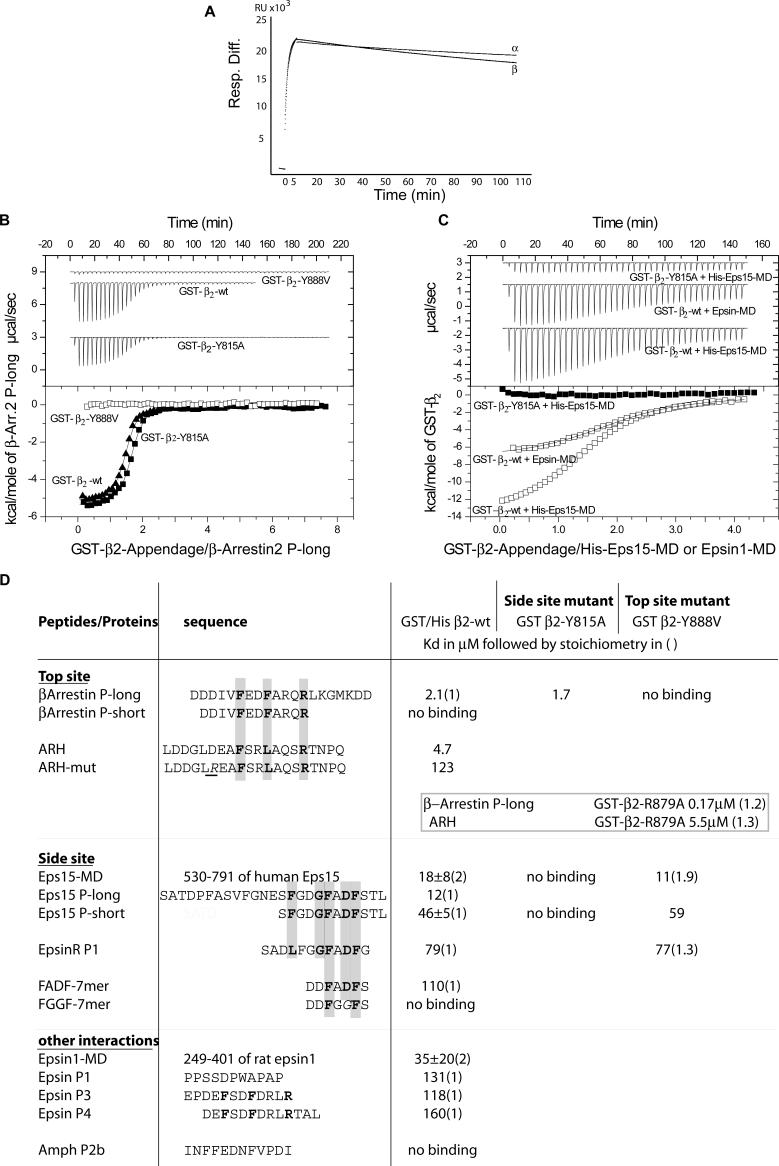
High Avidity Interactions of Eps15 for Clustered Appendages, yet Low Affinity Interactions of Ligands with Isolated Appendages (A) Surface plasmon resonance measurements of Eps15-MD binding α and β-appendages. Note that the protein binds, but does not come off even after extensive washing. (B) Affinities of β-arrestin P-long (see D) for wild-type β2-appendage and mutants measured by ITC. (C) Affinity measurement of protein domains with β2-appendages. (D) Table of peptides used, highlighting possible motifs, and a summary of affinities.

We started to examine the on- and off-rates for ligand interactions with appendages using surface plasmon resonance (SPR) where α- or β-appendages were coupled to chips and the motif domain of Eps15 (Eps15-MD, which covers the unstructured region, residues 530–791) was allowed to interact (Figure 3). In both cases the Eps15-MD bound so tightly that the off-rate cannot be measured as it takes more than 1 h to significantly reduce the binding (Figure 3A). This is also seen from empirical observations that in pull-downs using agarose bead-bound appendage domains most ligands do not wash off easily even after very extensive periods (up to 30 min, unpublished data). We assume that these observations of tight interactions are due to the presence of multiple appendage interaction sites in a single protein domain (for Eps15 we have previously identified four sites for α) [8]. Thus if the appendages are linked or bound to the same surface we observe a high avidity for the ligand interaction that is much stronger than the sum of the individual affinities [8]. This means that even a weak affinity can make a very significant contribution to a protein interaction, as long as there are multiple copies. This could be considered an artefactual observation, given the concentration of the appendages on a bead or on a SPR chip, but AP2 adaptors have two appendages and adaptor complexes are concentrated at sites of endocytosis and thus here they also present multiple appendages for interaction with ligands. Our observations of picomolar affinities for the monomeric Eps15-MD may not be accurate given that we do not observe an accurate off-rate, however this is likely to be an underestimate of the affinity of full-length Eps15 for AP2 as this protein is a dimer [26]. This is likely to have a physiological significance in giving stability to coated pit assembly, but this glue-like interaction also posits a significant problem for the progression of coated vesicle formation and the displacement of accessory proteins during the maturation of the coated pit.

### A Ligand Interaction Site on the β-Sandwich Domain of the β-Appendage

To understand appendage interactions we need a more detailed molecular description. By mass spectrometry interactions with Eps15, intersectin, RalBP1, and HivRevBP are still present in top site mutants and not with the side site mutant (unpublished data) and thus they interact predominantly with the side site. Knowing that Eps15 interacts primarily with the side site of the β-appendage (see also Figure 2B) we tested Eps15-MD (Figure 3C and 3D). We found that two appendages could interact with each copy of this domain with affinities of around 20 μM [8]. This interaction was abolished by a side site mutant and unaffected by a top site mutant (Figure 3C and 3D, note that we have used full-length Eps15 protein in Figure 2B). Thus we confirm a specific interaction of Eps15 with the side site of the β-appendage. To further narrow the interaction sequence we tested a number of peptides (previously made to test the interaction with the α-appendage) and found peptides that can interact with the β-appendage (Eps15 P-long and P-short). A mutant of the side site abolished the interaction (Figure 3D, Eps15 P-short).

**Figure 4:**
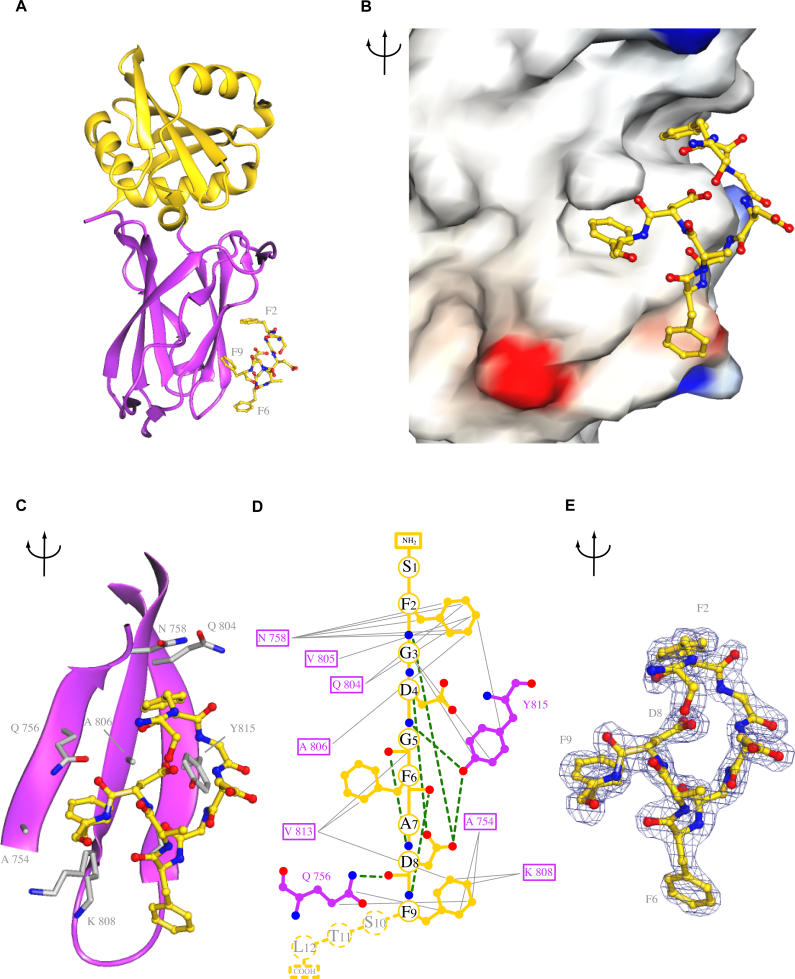
Eps15 Peptide Binds in a Tight Turn to the Side Site of the β2-Appendage (A) Ribbon diagram showing peptide in yellow bound to side site of the β-appendage. (B and C) The two principle Phe residues of the peptide bind in a groove. A single turn of a helix can also be seen for this peptide. (D) Peptide displayed as a linear chain showing hydrogen-bonding potential (green lines) and hydrophobic interactions (grey lines). A cluster of hydrogen-bonds in the peptide is consistent with the α-helical conformation in this region. (E) Density map for the peptide contoured at 1.24 σ.

We obtained co-crystals for Eps15 P-short peptide (SFGDGFADFSTL) with the β-appendage and see density for the complete peptide (Figure 4 and Table S1). The peptide forms a tight turn with the side-chains of F_2_ and F_9_ in a groove and that of F_6_ lying against a shallow pocket. The tight turn is formed by G_5_, and the psi and phi angles for this residue lie in a region of the Ramachandran plot permitted only for glycine residues. The turn is stabilised by the side chain of D_8_: it forms hydrogen bonds with the backbone nitrogen atoms of G_3_ and G_5_. The D_8_ side chain also forms a hydrogen-bond with Y815 of the appendage giving specificity to the peptide. The main chain carbonyl group of D_8_ also forms a hydrogen-bond with Q758. F_2_ is surrounded by N758, Q804, V805, A806 and Y815 where A806 is found in the base of the groove. F_6_ makes a hydrophobic interaction with V813. F_9_ projects into a shallow pocket lined by A754, Q756, K808, and V813. From F_6_ onwards, the peptide forms one turn of α-helix before the density fades. There were two β-appendage molecules in the asymmetric unit and consequently the peptide binding sites are in different crystallographic environments. The peptide we describe (that bound to molecule A) is sandwiched between the two molecules but is mostly bound to A. There are a few stabilising interactions with residues from B. We observe hydrogen bonds from the backbone nitrogen of chain B L819 and the ring nitrogen of chain B W714 to the side chain of D_4_ along with some other interactions via water molecules, as well as some interactions with the partially exposed F_6_. Molecule B has the Eps15 peptide in an identical position despite the peptide being exclusively solvent-exposed here. The density for this peptide is weaker here so it is possible that the binding site on this molecule was partially occupied (the K_D_ being 46 μM). However, we see density for two Fs in the same positions as F_2_ and F_9_ in the peptide bound to molecule A. As the density is weak it has not been possible to model this peptide unambiguously.

Mutagenesis of this site confirms that Y815 on the β-appendage is a critical residue presumably as it π stacks with F_2_ and H-bonds to D_8_. K808E also significantly weakens the interaction of amphiphysin, but has little effect on Eps15 as expected from the structure (Figure 2B). K759 is also close to the interaction site but is not involved in Eps15 peptide binding. K719 is a conserved residue at the distal end of the conserved patch and is not involved in peptide interactions nor does it affect the interaction of any ligands tested.

We made a peptide from epsinR that has sequence similarity to the Eps15 peptide. This epsinR peptide bound with an 80 μM affinity and a top site mutant does not reduce this affinity. Thus this is likely a side site interaction. To further examine the role of D_8_ in the interaction we made short peptides covering the FxDF sequence. An FADF 7mer peptide bound with an affinity of approximately 110 μM, and a mutation of the D to G gave no binding. Based on our structure we can propose that the FxDF peptide binds in a conformation similar to the α-helical turn in our Eps15 peptide. Taken together we think that F_9_ can be separated from the initial F_2_ by either a few residues and a tight turn (if there is a glycine present) but conceivably there could be more intervening residues, obviating the need for a glycine. Also the hydrophobic residues could easily be replaced by others of similar hydrophobicity. Thus the motif in Eps15 and epsinR is of the form [F/L]xxGFxDF but it could also be [F/L]x_n_[F/L]xDF, where *n* >3.

### Folding of β-Arrestin C-Terminal Peptide into an α-Helix on Interaction with the β-Appendage

**Figure 5:**
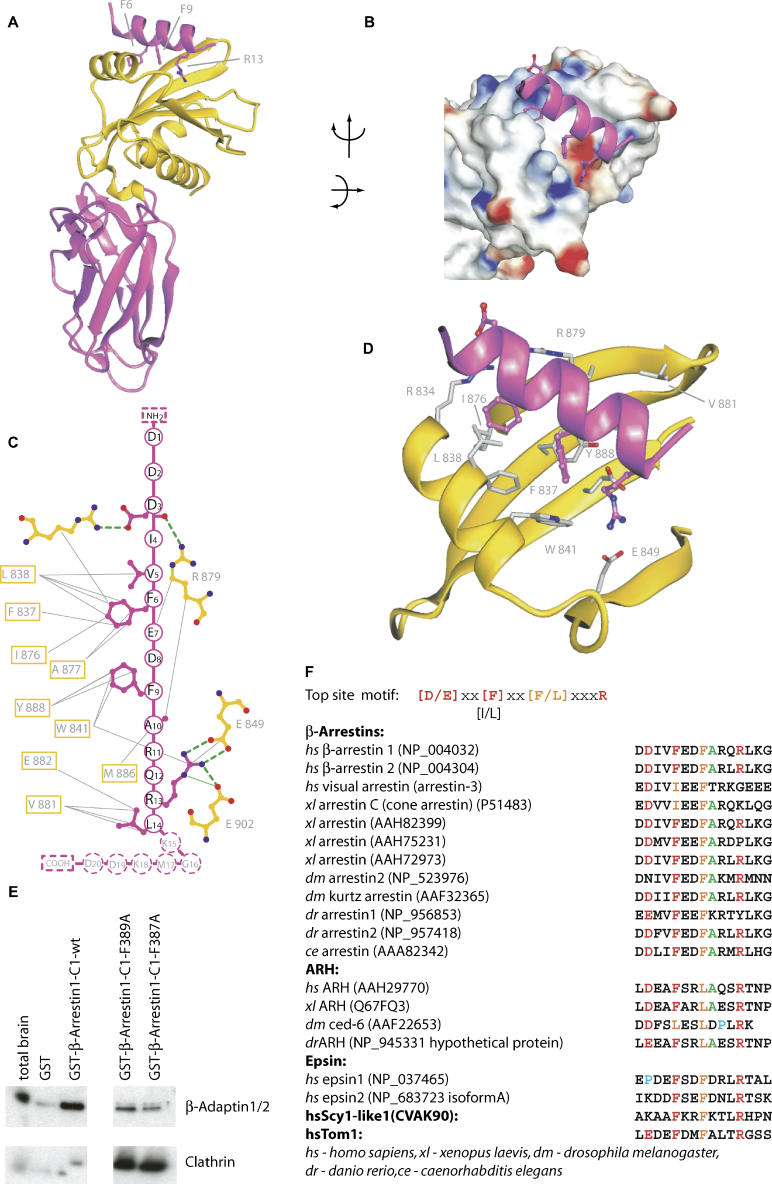
Peptide from the C-Terminus of β-Arrestin Folds as a Helix in a Groove on the Platform Sub-domain of the β2-Appendage (A) Ribbon diagram showing peptide in purple bound to top site of the platform sub-domain of the β-appendage as an α-helix. (B and D) The peptide binds into a groove with interacting residues of the peptide lining one side of the helix. The top of the groove is generally positive and D_3_ of the peptide is found here. F_6_ and F_9_ are in apolar environments and there is limited space for F_9_. R_13_ binds to the negatively charged patch of the groove. (C) Peptide displayed as a linear chain showing hydrogen-bonding potential (green lines) and hydrophobic interactions (grey lines). Hydrogen bonds within the α-helix of the peptide are not shown for clarity. The residues for which there is little density are dotted. (E) Mutagenesis of the Phe residues of β-arrestin DxxFxxFxxxR motif prevent the binding of AP2 complexes to GST-β-arrestin C1 in brain extracts. (F) Conservation of the interaction motif between arrestins and ARH and possible motifs from epsin and CVAK90.

Given the specificity of the β-arrestin interaction with the top site of β (see also [27]), we made peptides based on data from previous mutagenesis of the C-terminal tail of arrestin [22,27–29] and tested the interaction with the β-appendage by isothermal titration calorimetry (ITC). A β-arrestin peptide (P-long: DDDIVFEDFARQRLKGMKDD) bound with an affinity of approximately 2 μM and this was completely abolished by a top site mutant and not by a side site mutant of the β-appendage (Figure 3B and 3D). We previously thought that α- and β-top sites would interact with similar motifs, given the conservation of the site [13] but an amphiphysin peptide (Amph DNF 12mer) that binds with an affinity of 2.5 μM to the top site of α via an FxDxF motif, shows no binding to β (Figure 3D). Also β-arrestin P-long does not have a DxF or FxDxF motif found in top site interactors of the α-appendage, thus the binding modes must be different. To understand the specificity of the β-appendage top site we solved the crystal structure of β2-appendage in combination with β-arrestin P-long (Figure 5 and Table S1).

β-Arrestin P-long peptide (DDDIVFEDFARQRLKGMKDD) is derived from the C-terminus of β-arrestin and in crystal structures of β-arrestin 1 and 2 [28,30] this region packs against the N-terminal sub-domain of the arrestin molecule in an extended conformation. By circular dichroism this peptide, in solution, is not helical (unpublished data) but in our β-appendage crystal structure the β-arrestin peptide is folded as an α-helix in a groove between α-helix 1 and the antiparallel β-sheet of the platform sub-domain (Figure 5A). Thus the peptide folds on appendage binding. A similar observation is seen for an ARH peptide bound to the β-appendage in a paper just published [25]. In our structure of the β-appendage:β-arrestin P-long co-complex, residues D_3_–R_13_ were clearly visible and the induced helix breaks down after the arginine residue. The main interacting residues on the helix are D_3_xxF_6_xxF_9_xxxR_13_. All these residues lie on one side of the helix. Our density for D_3_ is not well defined but the side-chain appears to make hydrogen bonds with R834 and R879. F_6_ is in a hydrophobic environment created by F837, L838, and I876. F_9_ projects into a hydrophobic space lined by W841 and Y888. It shows π stacking with Y888 while W841 lies at the base of this groove. Mutation of W841 is likely to disrupt the local environment. The density at the base of F_9_ is not well defined, indicating some flexibility. Indeed, in the analogous sequence from ARH, an L occupies this position (see also [25]). The guanidino group of R_13_ forms hydrogen bonds with E849 and E902. L_14_ of the peptide, where the helix breaks, interacts with V881. The core motif, as revealed by the structure, is DxxFxxFxxxR, with the additional constraint that this must be found in the context of an α-helix, unlike the unfolded nature of peptide interactions known for other appendages. It is now evident from the structure why the mutation Y888V inhibits β-arrestin binding (see Figure 2B). Other key interaction residues are E902 and E849 and have been shown to be important in previous studies by others [27,29]. We note that R879 moves substantially between the unbound and the β-arrestin peptide-bound structure and a R879A mutation results in a 10-fold stronger interaction with β-arrestin (Figure 2B and 3C). The mutant is missing a hydrogen bond to the peptide via the terminal guanidino group of R879. Replacement of this side-chain may provide more space for a tighter interaction of the peptide with the groove. We shortened the arrestin peptide and although P-short has the core motif, it does not bind (Figure 3D), possibly because the peptide is too short to form a helix. The disruption of the helix may have caused previous difficulties in mapping the precise β-adaptin interacting residues on β-arrestin [22,27]. The interaction motif is conserved in β-arrestins 1 and 2 across different species (Figure 5F). Weak binding to AP2 has been reported for (bovine) visual arrestin [22] even though the motif is not completely conserved (Figure 5F). This motif is also found in the C-terminus of ARH, although F_9_ is here an L. A peptide covering this motif in ARH binds with a similar affinity to arrestin (Figure 3D). By looking at the homology of this motif across species it is likely to have a more generic form of [E/D]xxFxx[F/L]xxxR. An A frequently follows the second F and the initial D is often found with a cluster of acidic residues, perhaps aiding interaction with the generally basic potential of the surface of the β-appendage in this region. Given that the necessity of the initial D is not clear from the structure, we tested a mutant of this residue in ARH for the affinity of its interaction with β-appendage. The D to R mutant has a 30-fold lower affinity (Figure 3D and [25]). Compared to wild-type appendage, the R879A mutant of the β-appendage, which bound the β-arrestin peptide with higher affinity, does not have a different affinity for ARH (likely due to a serine after the first F in ARH being easier to accommodate near the interaction groove).

Scy1-like1 (CVAK90) has an FxxFxxxR sequence and Dab2/Disabled2 has an FxxFxxRQ. We find these proteins by mass spectrometry with the side site mutant but not with the top site mutant. We confirmed that Dab2/Disabled2 binds to the top site by Western blotting (unpublished data). Tom1, found as a β-appendage interactor in HeLa cells, also has an EDxFxxFxxxR. Epsin1 binding to β-appendage is weakened by Y888V top site mutant (Figure 2B). We found a possible interaction motif in epsin1/2 (see Figure 5F) and made a peptide covering this motif (see also [25]). This peptide (epsin1 P3) has a similar low affinity to β-appendage as the ARH peptide with its D mutated (118 / 123 μM, see Figure 3D). With this low affinity there must be a second mode of interaction required for efficient epsin1 binding to the β-appendage.

Based on the structure we made mutants of the β-arrestin C-terminus, F388A and F391A. In our structure the equivalent residues of the peptide both bind into the extended hydrophobic groove on the β2-appendage. Both of these mutants severely disrupt binding to the β-appendage (Figure 5E), confirming our structural observations. R395 of β-arrestin has previously been shown to be important for the interaction [22,27]. While the β-arrestin C-terminus binds to both clathrin and AP2 adaptors, we observe the clathrin interaction is increased when adaptor interactions are inhibited. Thus these interactions are likely to be mutually exclusive and indeed the close proximity of the clathrin terminal domain interaction motif (LIEFD, [31]) and adaptor binding motifs on β-arrestin (there are seven intervening residues) will result in stearic hindrance.

### Linking Clathrin Assembly to Adaptor Clustering

Many clathrin and adaptor interaction sites on accessory proteins overlap (including those of β-arrestin above), leading to the possibility that accessory proteins may swap from the AP2 appendage hub to the clathrin hub during coated pit assembly. However one must deal with the high avidity of accessory protein interactions with concentrated AP2 complexes (see Figure 3A). Normally a higher affinity interaction is required to displace a previous one but given that in a coated pit clathrin self-polymerises (at a critical concentration), even low affinity interactions of clathrin with the appendages may be sufficient to displace higher affinity ligands (see below for a more detailed discussion).

**Figure 6:**
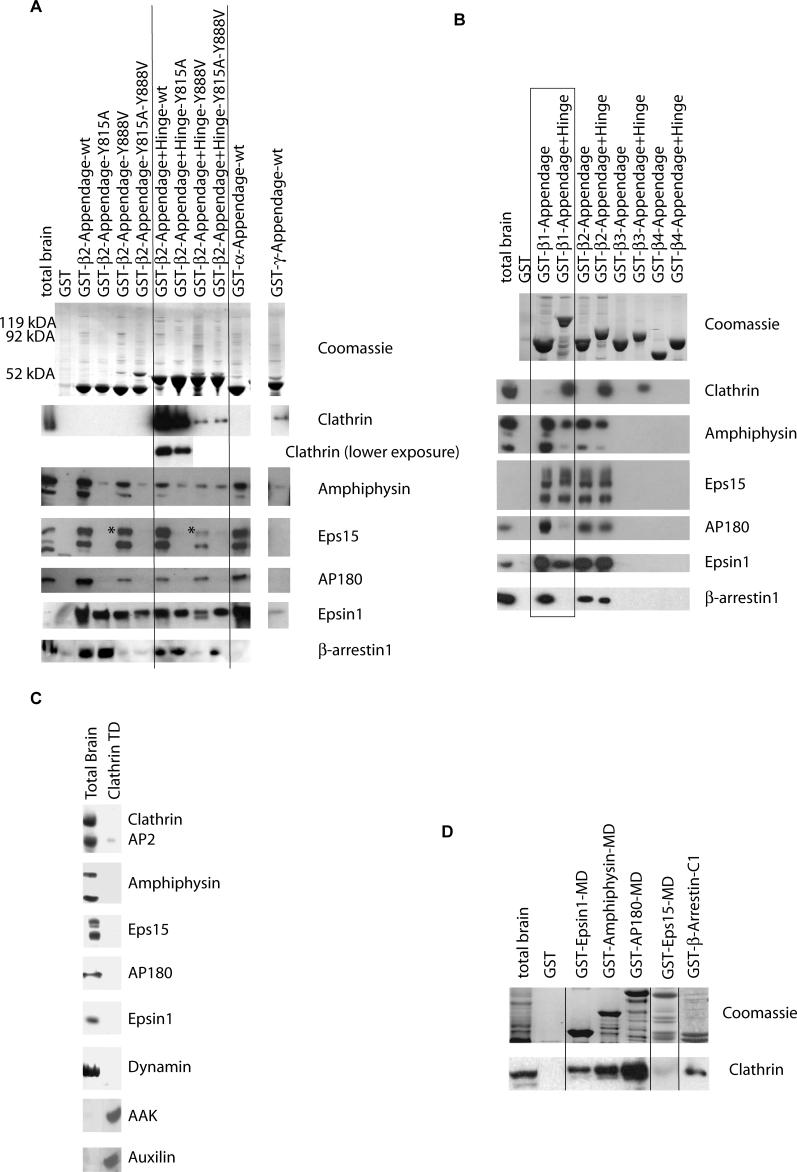
Introducing the Clathrin Hub (A) Displacement of β-appendage interactors with clathrin binding. A comparison of protein interactors with the β-appendage +/− the connecting linker to the core of the adaptor complex (the hinge domain) is made. Rat brain extract was used. The asterisks point to a reduced binding of Eps15 to the Y888V mutant only when clathrin is present. It can be noted that β-arrestin is not enriched in the pull-downs over total lysates (where 1% of the volume used for the pull-down is loaded) and given the high affinity of arrestins for the β-appendage we conclude that only a small percentage of the arrestin is in the “open” conformation necessary for β-appendage binding. Amphiphysin is also not enriched, but it has a much lower affinity for the β-appendage. (B) β1 appendage+hinge binding to clathrin results in reduced accessory protein interactions. Appendage and appendage+hinge proteins from β1 to β4 adaptins and their interactors are compared. We found no specific bands by mass spectrometry that interact with β3 and β4 in these experiments (apart from chaperones) See Figure S3 for β1–4 appendage homologies. (C) Clustered clathrin N-terminal domain interactions with accessory proteins. The GST-β-propeller domain has been bound to sepharose beads and thus we should especially detect proteins that can bind to clathrin via multiple interactions. (D) Clathrin binding of MDs from various accessory proteins except Eps15. GST-MDs on beads were incubated with rat brain extract and clathrin was detected by Western blotting.

We previously reported that clathrin polymerises around the β2-appendage+hinge causing displacement of other interaction partners [13]. The flexible “hinge” sequence between the core of the adaptor complex and the appendage has multiple clathrin terminal domain interaction motifs. We also showed that clathrin interacts with the top site of the β-appendage likely with a site C-terminal to the clathrin terminal domain. Given that we now know that most ligands of β bind to the side site we made a more extensive assessment of the role of clathrin in ligand displacement (Figure 6). In pull-down experiments (Figure 6A) clathrin is highly enriched on the β2-appendage+hinge protein but is not visible with the appendage alone in line with clathrin interactions with both the appendage and the hinge. A mutant of the top site, Y888V, reduces clathrin-binding effectively (in accordance with [13]). A mutant of the side site had a much weaker effect on clathrin binding (see lower exposure of clathrin in Figure 6A) showing that the predominant clathrin interaction with the appendage is with the top site. In a recent paper it was concluded that clathrin interacts with the side site, but in this study they have not examined clathrin interactions with the top site mutants or taken into account the previously published information, and thus their conclusion on clathrin displacement are misleading [25]. When clathrin is bound to the appendage+hinge then there is a reduction of β-arrestin, amphiphysin, and AP180 binding. The displacement of Eps15 and epsin was more variable (see epsin1 binding to β1-appendage+hinge in Figure 6B) and is likely due to the high avidity and multiple interaction sites of these proteins for β2-appendages (see also Figure 3C and 3D). In Figure 6B the effect of clathrin interaction with β1 is shown and there is a significant direct displacement of the top site interactor β-arrestin and a reduced interaction of AP180, amphiphysin and epsin1. This again points to the role of clathrin in the displacement of accessory proteins as coated pits are formed.

Many accessory proteins have both adaptor-appendage and clathrin terminal domain interaction motifs and thus we used GST-clathrin terminal domain bound to sepharose beads in pull downs from brain extract. This should allow us to find proteins that bind with a high avidity to multiple terminal domains. Eps15 and dynamin have no clathrin interaction motifs. AP180, amphiphysin and epsin1, which all have clathrin interaction motifs, show no visible interaction with clustered terminal domains (Figure 6C). This was a rather unexpected finding. This shows that the affinity between clathrin terminal domains and most accessory proteins is not high enough to be detected in pull-downs and thus clathrin is unlikely to recruit these proteins to sites of endocytosis. We then did the reverse experiment and used motif domains of accessory proteins clustered on beads and found clathrin does bind efficiently (Figure 6D). This is likely due to the stabilization of clathrin through self-interactions. Thus these proteins have the appropriate properties to recruit clathrin when clustered at nascent pits.

In contrast to the above we do observe a higher affinity of auxilin and adaptor-associated kinase (AAK) on clustered GST-clathrin terminal domains (Figure 6C). These proteins are likely to be recruited primarily by clathrin over adaptors. Auxilin is already known to be involved in uncoating and we would also propose that AAK is likely a kinase working at this same stage. AAK may function at multiple stages given that it has previously been shown to phosphorylate the μ2 subunit of the AP2 adaptor complex resulting in efficient cargo recognition [32].

We conclude that AP2 is the major accessory protein recruiting hub in early stages of clathrin-coated pit assembly.

### Changing Hubs

**Figure 7:**
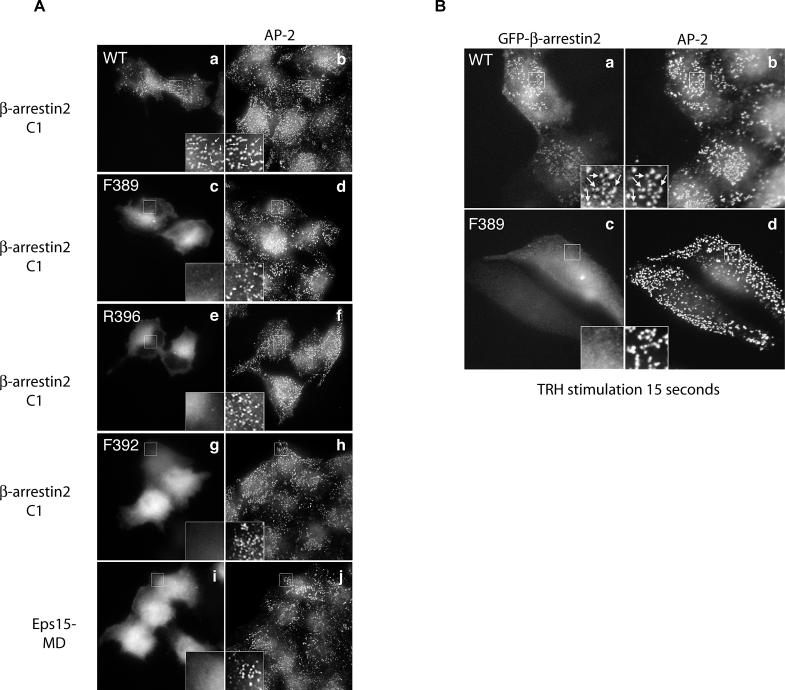
Selection of Partners to Remain during Coated Pit Maturation; β-Arrestin but not Eps15 Co-Localise with AP2 Adaptors in CCPs (A) GFP-βarrestin2 C-terminal domain co-localisation with AP2 adaptors while mutants are cytoplasmic. GFP-Eps15-MD is cytoplasmic. (B) Full length GFP-β-arrestin2 co-localisation with AP2 adaptor following stimulation of GPCR receptors with TRH for 15s. A mutant of β2-appendage interaction does not localise to coated pits.

To look at the behaviour of β-appendage interactors in vivo (Figure 7), we chose β-arrestin (which binds directly to either adaptors or clathrin) and Eps15 (which only binds to adaptors). When expressed as GFP-fusion proteins, the C-terminal clathrin/adaptor interacting domain of β-arrestin2 constitutively targets to CCPs as shown by their co-localisation with AP2 adaptors (Figure 7A, a and b). This co-localisation was completely lost when the residues in β-arrestin shown to be required for efficient binding to β-appendage were mutated to alanines (F_6_, F_9_ and R_13_ in our β-arrestin P-long peptide) (Figure 7A, c–h). In contrast to the C-terminal domain, full-length β-arrestin only co-localises with AP2 after GPCR receptor activation [33]. Full-length β-arrestin2 translocates to coated pits on receptor activation, but an F389 mutant (F_6_ in our peptide) does not co-localise (Figure 7B). We would conclude that β-arrestin is recruited into coated pits via the AP2 β2 appendage, as a mutant of this interaction abolishes it. Based on the competition data above (Figure 5E) we would suggest that once β-arrestin is in the coated pit it is likely maintained there by changing its hub affiliation from AP2 to clathrin. In clathrin-coated vesicles from both liver and brain, APs are under-represented compared to clathrin [15], and thus the terminal domains will not be fully occupied and are free to accommodate the displaced β-arrestin. In in vitro pull-downs of GST-β2-appendage+hinge there is not an excess of clathrin (see Coomassie staining in Figure 6A and 6B) and thus β-arrestin is displaced. In contrast GFP-Eps15-MD domain is cytoplasmic and is not enriched in coated pits like β-arrestin. Eps15 has no interaction with clathrin and must be displaced as clathrin assembles. Given that it cannot rebind to clathrin it will be pushed to the edge of coated pits where free adaptors are being recruited [8,13,34]. This concentration of Eps15 at the edge of coated pits has been previously observed by electron microscopy [35] and is not enriched enough to be visible in immunofluorescence. Thus despite the higher avidity of the motif domain of Eps15 for AP2, compared to β-arrestin which has only one AP2 interaction site, it is not concentrated in clathrin-coated structures (Figure 7A).

Our data do not agree with others who claim that clathrin binds to the side site and thus ligands of the top site can remain in coated pits during maturation [25]. We conclude that changing hubs is the main reason for the maintenance of accessory proteins and adaptors during coated pit maturation. Thus β-arrestin is displaced from the AP2 hub by clathrin, but is able to rebind to the excess of clathrin terminal domains found in coated pits.

## Discussion

Our structural, proteomic and mutagenesis data show that endocytic proteins that bind predominantly to the top site of the α-appendage also interact predominantly with the side site of the β-appendage via different motifs. This leaves space for yet other proteins to interact with the top site of the β-appendage and the side site of the α-appendage and thus the AP2 appendages can initially be considered as scaffolds for protein assembly. It is interesting to note that: a) we can no longer assume that only unstructured linear peptide motifs are bound by appendages; b) we can no longer assume from site conservation that we can guess the binding motif; and, c) we can now assume that multiple regions of all appendage domains are used by interactors. There are 2 main sub-domains to most appendages (for review of appendages in COP and clathrin coat components and in GGAs see [3]) and it appears that each of these sub-domains has an independent interaction site for ligands. For the α-appendage both interaction sites can be used by the same protein to increase its affinity substantially while still remaining readily reversible [8] and this is also likely true for many β-appendage ligands given that mutations of either site reduce binding (Figure 2B).

### The β-Appendage Top and Side Sites

So far appendage domains have been shown to bind to unstructured linear peptides in generally unfolded regions of proteins which we call motif domains (MDs). We and others now show that the top site of the β-appendage is different in that it confers an α-helix conformation to β-arrestin and ARH peptides (see Figure 5 and [25]). Thus not only are there charge and hydrophobic components to the interaction but extra specificity is conferred by the requirement that the sequence folds into a helix with the interacting residues on one face of the helix. The C-terminal region of β-arrestin that contains the β-appendage binding motif is poorly structured in the β-arrestin structure and the critical Phe residues are pointing towards the core structure [36]. Thus it is likely that some switch is needed to reveal the sequence to the β-appendage for recruitment to sites of endocytosis. While we show that β-arrestins and ARH can interact using a helix motif of the form DxxFxx[F/L]xxxR we must also note that the conserved interaction surface could be used for other modes of peptide interaction. Also, the R879A mutant of the β-appendage binds to an arrestin peptide with a higher affinity than wild-type and thus the interaction is not optimised for the highest affinity. This is often observed in nature where there is a balance of affinity versus specificity.

The side site on the β-sandwich sub-domain is in a different location to the side site on α, γ and GGA appendages. It is centred around Y815 which forms part of a hydrophobic groove. In the structure of Eps15 P-short bound to the β-appendage this hydrophobic groove is occupied by an F followed by a tight turn of the peptide and a critical DF sequence. As with the top site of α-appendage there may be many possible sequences that can occupy this site, giving the opportunity for many different ligands to bind to this site on the appendage.

We still do not know why two different appendages are required but given that the same set of proteins are interacting with the top of α and the side of β, we can suggest there may be cooperativity in the interactions of both appendages with ligands.

### Building the Endocytic Interactome

**Figure 8:**
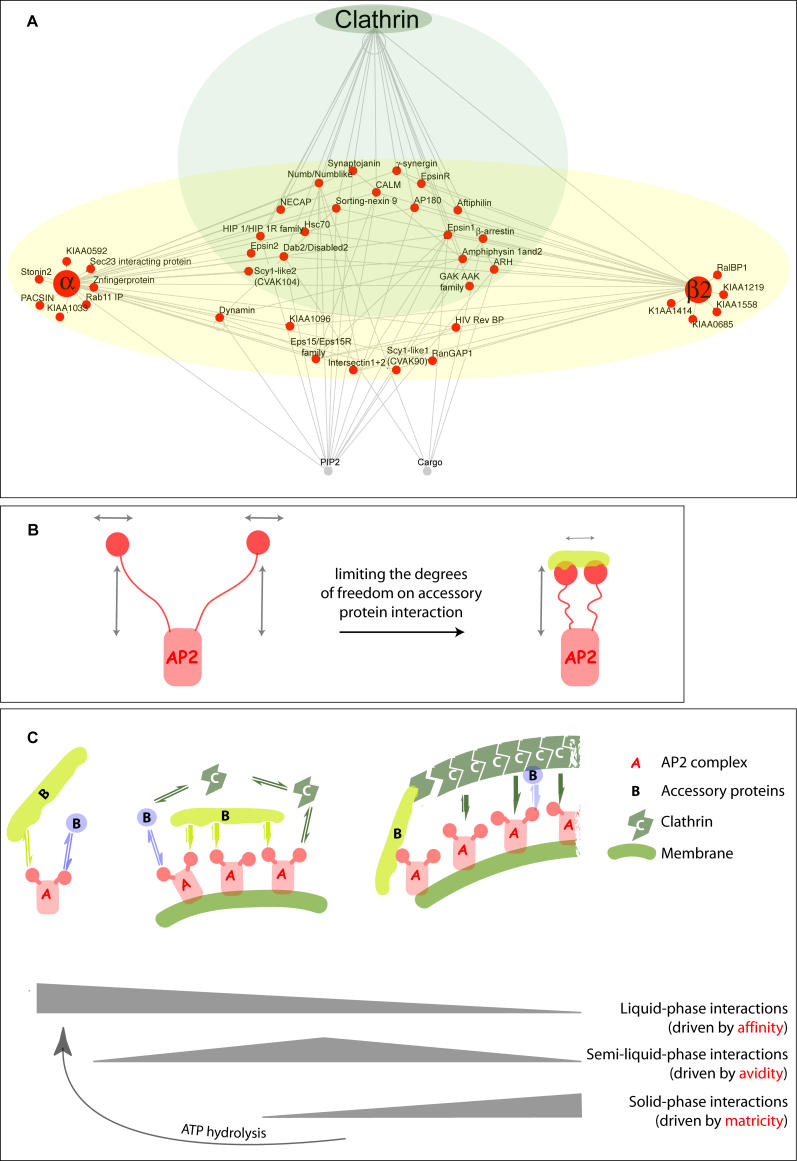
Network Dynamics (A) Network of protein interactions of AP2 appendages and clathrin. The α- and β2-appendage interaction data are from our mass spectrometry analysis and from the literature. The AP2 hub (yellow shaded area) is made up of 2-sub-hubs that increase the interactome repertoire (side arms) but for the majority of proteins they increase the interaction avidity (central circle). As the network matures and clathrin is recruited, many of the previous interactors become clathrin interactors (green shaded area). Of the remaining proteins we only know of a few that do not bind to clathrin, while others have simply not been tested. (B) Limiting the mobility of appendage domains by interactions of both domains with the same accessory protein. (C) The changing environment of adaptor protein complexes, A, in CCV formation, showing the gradual movement from simple affinity based interactions with accessory proteins, B, to avidity based interactions, once adaptors are recruited to the membrane. As the coated pit matures and clathrin, C, polymerises into a matrix then accessory proteins that cannot bind directly to clathrin are displaced to the edge where the pit is still growing. ATP hydrolysis is needed to depolymerise clathrin and re-prime the system.

β1 and β2 adaptors are highly homologous, while β3 has weak homology but no clear conservation of known interaction sites. β4 has similar weak homology and only has the platform sub-domain with clear conservation of the residues in this site (see Figure S3). By Coomassie gel analysis, we did not see major interaction partners for β3 and β4 (Figure 6B). Thus the interactors have lower affinity and perhaps one needs the correct combinations of appendages as found in AP3 and AP4 complexes to get higher avidity interactions. One can propose that these appendages are not used to aggregate cargo by cross-linking and thus the avidity effects we see with accessory protein interactions need not exist. It could also be that they are not directly involved in CCV formation but clathrin does bind to the β3-appendage+hinge protein (Figure 6B). To understand the interactions of AP1 and AP2 complexes we should also consider the network of interactors with both appendages and with clathrin. In Figure 8 we plot an updated network for the α- and β2 appendages (Figure 8A). While these diagrams do not give information on affinities and competition between ligands, we do begin to see the importance of knowing the context in which each protein operates. Given the concentrations of protein interactions on α- and β-appendages it is easy to understand why over-expression of appendage interacting domains from almost any protein will inhibit CCV formation.

Understanding CCV formation as a network of protein interactions can help us not only simplify the complex interactomes into core and peripheral parts, but it also helps us design our experiments. In a network each interaction of necessity must be of relatively low affinity if the network is to be dynamic. The meta-stable nature of complexes formed allows the network to evolve from one hub to another with directionality imposed by steps that are not readily reversible. In this view of CCP assembly, over-expression of many proteins within the network may well inhibit endocytosis while over-expression of a central hub in the process should have no effect. Likewise depletion of the hub proteins should inhibit while depletion of the more peripheral node proteins will in general be less dramatic. Thus the position of a protein in a network is an important consideration when adding GFP tags to proteins to probe their function. Additionally, keeping the levels of over-expressed protein close to those found for endogenous proteins is important, if a dominant-negative effect is not sought. The network of interactions also provides many points of contact with other cellular processes (like exocytosis and signalling pathways) and the dynamic nature provides many avenues to modulate endocytosis.

Our data raise questions as to the purpose of two different appendages in each adaptor complex. There are several consequences: a) it increases the range of proteins that can be bound to the AP complexes; b) if the same protein is bound to both appendages then this increases the avidity of the interaction; c) the multiple independent interaction sites on appendages provide an increased specificity to the interactions; and, d) the use of two appendages binding to one ligand should limit the mobility of the appendages in space (Figure 8B). Thus when linked, the flexible appendages will be constrained to mobility primarily in one plane, making it more likely that the ligands will be recruited back to the core of the adaptor complex.

### How CCV Endocytosis Works

We propose the following sequence of events for coated pit maturation (Figure 8C). The coincidence of multiple activated cargo receptors and PtdInsPs in the membrane results in the accumulation of adaptor complexes. Once adaptors are in close proximity to each other they become stabilised by cross-linkages using accessory proteins with multiple interaction sites for adaptor appendage domains. Many of these accessory proteins in turn recruit clathrin, and once this is concentrated at the site of the nascent vesicle it self-assembles. At this point the interactions between clathrin and the adaptors are no longer strictly “liquid-phase” and thus affinity measurements and dissociation constants do not accurately reflect the dynamics of the system. The clathrin coat makes a pseudo “solid-phase” interaction with the adaptor proteins, thus displacing the high avidity accessory protein interactions with much weaker clathrin:adaptor interactions. This means that off-rates for clathrin monomers in the coat are dramatically reduced thereby increasing the effective affinity for adaptors. This is quite different from the avidity effects we observe with accessory protein interactions where the off-rate should not significantly change. A dynamic instability in the process thus favours a forward direction only in the presence of sufficient cargo and the ultimate energy input is at the final stage of vesicle scission (GTP hydrolysis) and uncoating, where ATP is used to disassemble the clathrin coat enabling a return to liquid-phase affinities. Similarly it appears that ATP hydrolysis occurs in membrane fusion only after fusion has taken place [37] and this again is likely to give a dynamic instability until late in the process.

Thus two basic principles are deduced: 1) High avidity interactions of accessory proteins are replaced with what are apparently the weak interactions of the clathrin coat with adaptors. However these “matricity”-driven interactions are stable because the “off-rate” of clathrin monomers is now not a major factor. Hydrolysis of ATP displaces the clathrin coat thus re-priming the system. 2) Looking at CCV formation from an energetic point of view it might appear that the process is being pulled forward from the end point, where ATP hydrolysis is used to re-prime the clathrin coat protein. This makes good sense as the system has been designed with built-in instability and the point of no return is very late in the process. Thus initially low affinity (and therefore readily reversible) interactions between cargo and adaptors, between adaptors and accessory proteins, and between accessory proteins and clathrin, are used to build the network. Gradually the network becomes more stable and only then does it become necessary to utilize the energy from ATP hydrolysis to go to the next stage, and at this point CCV formation has already occurred.

## Materials and Methods

### Constructs.

GST-mouse-α-appendage (α-ear_L_695–983), GST-human-β2-appendage (700–937), 6× His-human-β2-appendage (700–937), GST-human-β2-appendage+hinge (616–937), GST-mouse-β1-appendage (707–943), GST-mouse-β1-appendage+hinge (512–943), GST-human-β3-appendage (853-1094), GST-human-β3-appendage+hinge (810-1094), GST-human-β4-appendage (570–739) (AHWAT to end), GST-human-β3-appendage+hinge (535–739) (SPKSD to end), and GST-mouse-γ-appendage (E3, 704–822) were expressed in the BL21 strain of E. coli. All proteins used for ITC and biochemistry were purified via affinity resins, Q-sepharose and gel filtration before use. For ITC experiments, thrombin cleaved α and 6× Hisβ2 or GSTβ2 were used after purification by affinity resins and gel filtration. Mutations were made by PCR mutagenesis and constructs were sequenced. All the key α-appendage mutants are well folded as monitored by the stoichiometry of binding in ITC experiments but the β-appendage mutants are less stable and precipitate over time.

6× His human Eps15-MD (530–791) (STSSSE…GKRSI) was cloned into EcoR1/Not1 sites of pET28c and a GST version was cloned into same sites of pGex4T2. GST rat epsin1-MD (249–401) (TGGKE…DTEPD) was cloned into EcoR1/Not1 sites of pGex4T2 and thrombin cleaved for ITC measurements.

GST human-β-arrestin2-C1 (317–410) and full length GST bovine-β-arrestin2 were used. GST rat AP180-MD (516–915) (ATAPS…IKDFL) was cloned into ER1/Not1 sites of pGex4T2. The GFP-tagged constructs were derived from rat β-arrestin2, the C-terminal tail fragment C1 (317–410) and mutations F389A, F392A, and R396A were made by PCR and constructs were sequenced.

GST rat Amph1-MD (1–390) (start… WTTSTD) was cloned into the ER1/Not1 sites of pGex4T1. Has DNF, DPF, and WxxW adaptor binding sites and the N-terminal BAR domain which will dimerise the protein.

GST bovine clathrin terminal domain residues 1–363 were used.

### Fishing in HeLa cell extracts with GST-appendages.

HeLa cells (1 × 10^8^ cells) were trypsinised and washed in 150 mM NaCl, 20 mM HEPES [pH7.4] with 2 mM DTT and protease inhibitors. Cells were solubilised with NP40 (not to disrupt the nuclei) and debris was pelleted. For interaction experiments 0.1% Tx-100 was added to 0.6 ml of this extract + 30–100 μg of GST fusion-protein + glutathione-sepharose beads, incubated for 1 h at 4 °C and then the bead bound proteins were washed 4× with the same buffer with Tx-100.

### Mass spectrometry analysis.

Peptides of in-gel trypsin digested protein bands were separated by liquid chromatography on a reverse phase C18 column (150 × 0.075 mm i.d., flow rate 0.15 μl/min). The eluate was introduced directly into a Q-STAR hybrid tandem mass spectrometer (MDS Sciex, Concord, Ontario, Canada). The spectra were searched against a NCBI non-redundant database with MASCOT MS/MS Ions search (http://www.matrixscience.com). For protein with a low number of peptides we have confirmed their identity by searching the PeptideSearch nrdb database using sequence tags from our data. Proteins in the same molecular range as the GST-appendages were sequenced from pull-downs where the appendages were cross-linked to beads (Affigel 10) to reduce the signal from the pull-down proteins.

### Crystallography and structure determination.

6× His-tagged β-appendage was expressed by overnight induction in BL21 pLysS cells. Protein in 20 mM HEPES, 150 mM NaCl, and 2 mM DTT was purified by passage over a Ni-NTA column after which the 6× His tag was cleaved off by a 2.5-h incubation with thrombin. The protein was subsequently purified further by passage over a Q sepharose column and a gel filtration column. Protein was concentrated to approximately 4 mM and flash frozen in liquid nitrogen for long-term storage. The crystal of the co-complex of β-appendage and β-arrestin P-long was obtained by vapour diffusion using sitting droplets, against a reservoir containing 0.2 M magnesium formate, 20% PEG 3350. Droplets were made by mixing 100 nl β-appendage:peptide mix (1.11:1.88 mM) with 100 nl of the reservoir. A solitary crystal grew over a period of a few weeks. Despite exhaustive attempts, crystals could not be regenerated. The crystal was cryoprotected by transfer to a buffer containing 0.2 M Mg formate and 30 % PEG3350. Data were collected at ID14–1 and ID14–4 at the European Synchrotron Radiation Facility, λ = 0.934 Å and λ = 0.980 Å. The crystal was split and highly anisotropic so we could only collect an incomplete dataset to 2.5Å. The crystal belonged to space group P2_1_2_1_2_1_, with unit cell dimensions 36.95, 35.37, 190.85Å.

Crystals of the β-appendage + Eps15 P-short peptide were grown by vapour diffusion, using sitting droplets, against a reservoir containing 2 M ammonium sulphate and 0.1 M sodium acetate [pH 4.5]. Droplets were made by mixing 1 μl of the protein-peptide mix (1:2 mM) with 1 μl of the reservoir solution. Crystals grew over a few months. The crystals were cryoprotected by transfer to a buffer containing 1.82 M (NH_4_)2SO_4_, 0.09 M NaOAc, 25 % glycerol. Crystals belonged to space group P2_1_2_1_2_1_ with unit cell dimensions 58.33, 71.97, 115.90 Å, with two molecules in the asymmetric unit. Data were collected at ID14–4 at the European Synchrotron Radiation Facility, λ = 0.979Å. Crystals were of excellent quality and a complete dataset was collected to 1.9 Å. Data were integrated using MOSFLM [38] and scaled using SCALA from the CCP4 suite of crystallographic software [39]. The structure was solved by molecular replacement using PHASER [40], using the structure of β2 adaptin chain A (PDB ID 1E42) as the search model. The model was corrected and completed using O and COOT and the structure was refined using REFMAC5 [41–43].

Coordinates and structure factors were deposited in the Protein Data Bank [44] with the accession codes 2iv8 (β-appendage with β-arrestin peptide in top site) and 2iv9 (β-appendage with Eps15 peptide in side site). Figures were generated using Aesop (Martin Noble, personal communication) and schematics of peptide-protein interactions were based on the output of LIGPLOT [45]. Surface potential maps were calculated using GRASP [46] and displayed in Aesop.

### Surface plasmon resonance.

SPR experiments were performed using a BIA2000 apparatus (BIAcore, Uppsala, Sweden). GST, GST-α, or GST-β2 were immobilized via amine coupling (according to manufacturer's instructions) on a CM5 (carboxymethyl) chip. Recombinant 6× His hEps15-MD was then injected at a concentration of 300 nM to saturate the surface. Running buffer was 20 mM HEPES, 150 mM NaCl, [pH 7.4]. Dissociation was measured over 7,000 s at a flow rate of 20 μl/min. Nonspecific binding was measured as 6× His hEps15-MD binding to the GST surface and subtracted from the experimental data. SPR data was analyzed using BIAevaluation software provided by the manufacturer.

### Isothermal titration calorimetry.

Binding of peptides and proteins to appendage domains was investigated by ITC [47] using a VP-ITC (MicroCal, Northampton, Massachusetts, United States). This technique allows us to calculate equilibrium association or dissociation constants for interactions. These are distinct from association and dissociation rate constants measured by surface plasmon resonance. Where we can accurately measure the concentrations of protein/peptides used we can get accurate values for the stoichiometry of interactions. Where proteins are not a single species due to degradation then the stoichiometry may be inaccurate but the affinity can still be measured if the concentration of the ligand in the syringe is accurate. All experiments were performed in 100 mM HEPES, 50 mM NaCl, 2 mM DTT [pH 7.4] at 10 °C and protein concentrations were determined by absorbance at 280 nm or at 257 nm. The peptides or proteins were injected from a syringe in 40–50 steps up to a 3–5 fold molar excess over the cell concentration. The cell contained 1.36 ml protein solution and typically the ligand was added in steps of 4–8 μl every 3.5 min. Concentrations were chosen so that the binding partners in the cell were at least 5-fold higher than the estimated dissociation constant, if possible. The ligands in the syringe were again at least 10-fold more concentrated. The heat of dilution of the ligand was subtracted from the data prior to fitting. Titration curves were fitted to the data using the ORIGIN program supplied by the manufacturer yielding the stoichiometry N, the binary association constant K_a_ (= K_d_^−1^) and the enthalpy of binding. The entropy of binding ΔS° was calculated from the relationship ΔG° = −RT lnK_a_ and the Gibbs-Helmholtz equation. Peptides were purchased at > 95 % purity from the Institute of Biomolecular Sciences (University of Southampton, United Kingdom) and weighed on an analytical balance and verified by measuring the OD280 or OD257. The resulting concentration errors are estimated to be < 10%. Unless otherwise stated the values for the stoichiometry, N, were within this error region around N = 1.

### Cells transfections and immunofluorescence.

HeLa cells (ATCC) were grown in DMEM supplemented by 10% foetal bovine serum (Invitrogen, Paisley, United Kingdom). Cells were transiently transfected with GFP constructs and AP2 was detected with monoclonal antibody 100.2 (Sigma, St. Louis, Missouri, United States). Thyrotropin releasing hormone (TRH) was from Sigma. For immunofluorescence HeLa cells on coverslips were used for immunofluorescence studies the day after transfection. Cells were washed in PBS and fixed in 3.7% paraformaldehyde 0.03 M sucrose for 30 min at 4 °C, then washed once in PBS and quenched in 50 mM NH_4_Cl in PBS. The cells were then incubated with primary antibodies in permeabilization buffer (PBS supplemented with 1mg/ml BSA and 0.1% triton X-100) for 45 min at room temperature. After two washes with PBS 1mg/ml BSA, cells were incubated for 45 min at room temperature in PBS 1 mg/ml BSA containing secondary antibodies. After two washes in permeabilization buffer and one in PBS, the cells were mounted on microscope slides in PBS-glycerol (50/50).

Samples were examined under an epifluorescence microscope (Leica, Wetzlar, Germany) with a cooled CCD camera (Micromax, Princeton Instruments Lurgan, United Kingdom). Images were acquired with MetaMorph (Universal Imaging, Downingtown, Pennsylvania, United States) and processed with MetaMorph, NIH image (http://rsb.info.nih.gov/nih-image) and Photoshop (Adobe Systems, San Jose, California, United States).

## Supporting Information

Figure S1 Fishing in Rat Brain/Liver Cytosol with GST-Appendages (1.9 MB TIF)

Figure S2 Schematics of Short Peptide Motifs and Domains in New Proteins Obtained in the Current Study (2.3 MB TIF)

Figure S3 Homologies between α and β1–4 Appendage Domains (6.1 MB TIF)

Table S1 Crystallographic Statistics (27 KB DOC)

### Accession Numbers

The PubMed (http://www.ncbi.nlm.nih.gov/entrez/query.fcgi) accession numbers for the genes and gene products presented in this paper are AAK (NP_055726), aftiphilin (Q6ULP2), amphiphysin1 (P49418), AP180 (NP_113916), ArfGAP1 (CAG30268), ARH (AAH29770), auxilin (BAA32318), α-adaptin (P18484), β2-adaptin (P63010), β-arrestin2 (P32121), CALM (AAB07762), clathrin (NP_004850), Dab2/Disabled2 (AAF05540), epsin1 (NP_037465), epsin2 (O95208), epsinR (Q14677), Eps15 (CAI13030), Eps15R (NP_067058), HivRevBP (NP_004495), Hsc70 (NP_006588), intersectin1 and 2 (NP_001001132, AAF63600), KIAA0196 (BAA12109), KIAA0592 (AAH82258), KIAA0685 (XP_217014), KIAA1033 (BAA82985), KIAA1096 (BAA12109), KIAA1219 (BAA86533), KIAA1414 (XP_343001), KIAA1558 (BAB13384), RalBP1 (AAK34942), RanGAP1 (NP_055805), Scy1-like1/CVAK90 (NP_065731), Scy1-like2/CVAK104 (NP_060458), Sec23 interacting protein p125 (Q9Y6Y8), sorting-nexin9/snx9 (NP_057308), pacsin/syndapin2 (AAH08037), γ-synergin (AAD49732), Tom1 (NP_005479), and Znfinger protein (NP_115765).

## Abbreviations

AAK: adaptor associated kinase
AP180: adaptor protein of 180 kDa
AP2: adaptor protein complex 2
ARH: autosomal recessive hypercholesterolemia
CCP: clathrin-coated pit
CCV: clathrin-coated vesicle
CVAK90: clathrin vesicle associated kinase of 90kDa
Eps15: epidermal growth factor receptor pathway substrate 15
GAK: cyclin G-associated kinase
LC-MS/MS: liquid chromatography tandem mass spectrometry
GST: glutathione s-transferase
LDL: low density lipoprotein
MDs: motif domains
PtdInsP: phosphatidyl inositol phosphate
TRH: thyrotropin releasing hormone
