# Supplementary material for: Role of the AP2 β-Appendage Hub in Recruiting Partners for Clathrin-Coated Vesicle Assembly
Source: PLoS Biol. 2006 Aug 15;4(9):e262. doi: 10.1371/journal.pbio.0040262 (PMC1540706; doi:10.1371/journal.pbio.0040262)
Supplement: Supplementary file 1 [file pbio.0040262.st001.doc]

# Table S1

**Crystallographic statistics**

**Side-site crystal:**

Data collection statistics

| Spacegroup | P2_1_2_1_2_1_ |
| --- | --- |
| Resolution (Å) | 45.31 - 1.9 (2.0 - 1.9) |
| Completeness (%) | 97.9 (89) |
| Multiplicity | 7.0 (6.3) |
| Average Intensity (I) | 3332 (255) |
| Sigma (σ) | 657.1 (260.9) |
| Mean I / σ | 5.1 (1.0) |
| Wilson Plot B (Å^2^) | 29.7 |
| Rmerge (%) | 9.5 (73.4) |
| Rmeas (%) | 10.3 (79.8) |

Refinement statistics

| R (%) | 21.39 |
| --- | --- |
| R_free_ (%) | 26.74 |
| Test set size (%) | 5.03 |
| <B> Å^2^ | 38.96 |
| N_reflections_ (N_free_) | 38599 (1849) |
| N_atoms_ (N_water_) | 4111 (290) |
|  |  |
| r.m.s. deviations from ideality |  |
| Bond lengths Å | 0.1 |
| Bond angles º | 1.219 |
|  |  |
| Number of Ramachandran violations | 0 |

**Top-site crystal:**

Data collection statistics

| Spacegroup | P2_1_2_1_2_1_ |
| --- | --- |
| Resolution (Å) | 95.35 - 2.5 (2.64 - 2.5) |
| Completeness (%) | 70.1 (68.7) |
| Multiplicity | 4.2 (4.4) |
| Average Intensity (I) | 908 (137) |
| Sigma (σ) | 114.3 (56) |
| Mean I / σ | 7.9 (2.4) |
| Wilson Plot B (Å^2^) | 49.9 |
| Rmerge (%) | 6.8 (30.8) |
| Rmeas (%) | 7.7 (34.6) |

Refinement statistics

| R (%) | 21.99 |
| --- | --- |
| R_free_ (%) | 36.86 |
| Test set size (%) | 4.5 |
| <B> Å^2^ | 33.859 |
| N_reflections_ (N_free_) | 4727 (215) |
| N_atoms_ (N_water_) | 2032 (16) |
|  |  |
| r.m.s. deviations from ideality |  |
| Bond lengths Å | 0.024 |
| Bond angles º | 2.246 |
|  |  |
| Number of Ramachandran violations | 0 |
